# Short-term quantitative CT changes in synchronous ground-glass nodules during immune checkpoint inhibitor therapy in patients with lung cancer

**DOI:** 10.3389/fimmu.2026.1810440

**Published:** 2026-06-24

**Authors:** Yuting Zheng, Shiqi Li, Tingting Guo, Qinyue Luo, Yimeng He, Mengting Huang, Heshui Shi, Xiaoyu Han

**Affiliations:** 1 Department of Radiology, Union Hospital, Tongji Medical College, Huazhong University of Science and Technology, Wuhan, China; 2 Hubei Provincial Clinical Research Center for Precision Radiology and Interventional Medicine, Wuhan, China; 3 Hubei Key Laboratory of Molecular Imaging, Wuhan, China

**Keywords:** immune checkpoint inhibitor, longitudinal changes, lung cancer, quantitative CT analysis, synchronous ground-glass nodule

## Abstract

**Background:**

Immune checkpoint inhibitors (ICIs) have improved survival in patients with lung cancer, leading to more frequent detection of synchronous ground-glass nodules (GGNs) during follow-up. This study aimed to evaluate short-term longitudinal quantitative CT changes of synchronous GGNs in lung cancer patients receiving ICI therapy.

**Methods:**

In this retrospective matched cohort study, we included 90 patients with lung cancers harboring 110 synchronous GGNs receiving 3–4 cycles of ICIs, and compared them with a matched cohort of incidentally detected pulmonary GGNs without any therapy. Baseline and follow-up chest CT scans were quantitatively analyzed to capture changes of diameter, volume, surface area, mass, mean CT attenuation, standard deviation of CT attenuation, solid component proportion, sphericity, energy and entropy. Volume change proportion (VCP) was assessed to define nodule response, using ± 25% as the threshold. Subgroup analyses were conducted according to nodule density and Lung-RADS category.

**Results:**

VCP showed that nodules in ICI group were more likely to show regression than those in the control group (23.6% *vs*. 1.8%, p< 0.001). Improvement in Lung-RADS category was also more common in the ICI group than in controls (8.2% *vs* 1.8%; p = 0.018). Compared with matched controls, nodules in the ICI group showed significantly lower monthly increases in diameter, volume, surface area and mass. Within the ICI group, nodules showed a significant reduction in diameter, while increases in mean CT value, standard deviation, solid component proportion, and entropy. In subgroup analyses, part-solid nodules (PSNs) showed larger volume reduction than pure GGNs, and nodules with Lung-RADS ≥ 4A showed a significant decrease in entropy.

**Conclusion:**

Among lung cancer patients receiving ICIs, synchronous GGNs exhibited attenuated longitudinal CT growth patterns, with favorable shifts in VCP and Lung-RADS categories, particularly in PSNs and nodules with Lung-RADS ≥ 4A.

## Introduction

Lung cancer is one of the most commonly diagnosed cancers and the leading cause of cancer death worldwide (1). Recently, the detection rate of early-stage lung adenocarcinomas manifesting as ground-glass nodules (GGNs) is increasing (2). Given the indolent nature of GGNs, it remains clinically challenging to avoid overtreatment while ensuring timely intervention for lesions with malignant potential (3, 4). This dilemma is further amplified in patients with multiple synchronous GGNs, particularly when lesions are distributed across different lobes, making surgical management technically demanding (5). Thus, longitudinal quantitative imaging-based assessment may help characterize temporal changes in GGNs.

Immune checkpoint inhibitors (ICIs) have been shown to improve long-term survival in patients with advanced non-small cell lung cancer (NSCLC) (6). In resectable NSCLC, ICIs have also demonstrated benefits in enhancing pathological response rates and clinical outcomes (7). However, GGNs are not therapeutic targets of ICIs, and their biological behavior differs substantially from that of advanced solid tumors. Studies have reported that GGNs are characterized by reduced metabolism activity and a less active immune microenvironment, suggesting that GGNs may respond poorly to ICI therapy (8, 9). Nevertheless, emerging evidence indicates that immune escape may occur at pre-invasive stages of cancer development, supporting the application of immune intervention in precancerous lung nodules to prevent them evolve into invasive diseases (10–12).

Recent prospective studies have explored the potential effects of immunotherapy on pulmonary GGNs (13–15). Cheng et al. (13) reported that among 36 patients with synchronous GGNs treated with sintilimab, the overall objective response rate was 13.9%, with no cases of progressive disease. Park et al. (14) reported that canakinumab induced nodule regression in 10 of 15 patients and might delay progression of persistent high-risk pulmonary nodules. Xu et al. (15) evaluated sintilimab in 21 lung cancer patients with synchronous GGNs, and found no significant change in mean nodule size during follow-up, although postoperative pathology revealed that 15.4% of GGNs achieved major pathological response (MPR). Collectively, these preliminary findings suggest that immunotherapy may hold promise for GGNs. However, these studies were limited by small sample cohorts, lack of control groups, and reliance on RECIST criteria, which may be insensitive for GGNs.

Therefore, we conducted a retrospective matched cohort study to quantitatively assess short-term longitudinal CT changes in synchronous GGNs in lung cancer patients receiving ICIs. By leveraging nodule-level matching and quantitative imaging features, this study aimed to characterize growth kinetics and imaging trajectories of GGNs during ICI exposure.

## Materials and methods

### Study population

**Figure 1:**
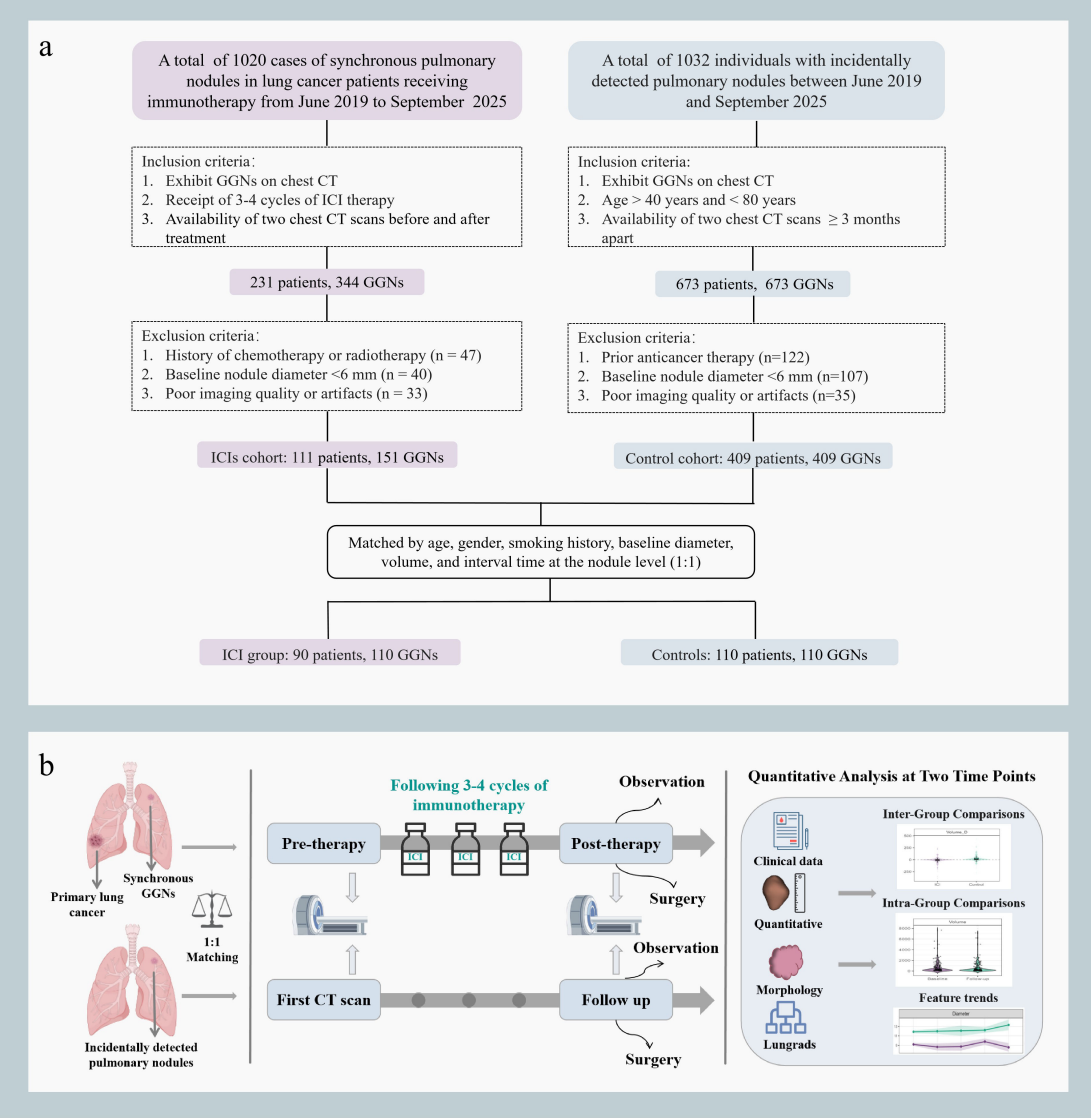
Patient inclusion and exclusion criteria **(a)**, and study flowchart **(b)**.

We retrospectively reviewed 1020 lung cancer patients with synchronous pulmonary nodules treated with ICIs at Wuhan Union Hospital from June 2019 to September 2025. The study design and patient selection were presented in **Figure 1**. The inclusion criteria were as follows: (1) exhibit GGNs on chest CT; (2) receipt of 3–4 cycles of ICI therapy, and (3) availability of two chest CT scans before and after treatment. Initially, 231 patients with 344 GGNs fulfilled the criteria. Patients were excluded for: (1) history of chemotherapy or radiotherapy (n=47); (2) baseline nodule diameter<6 mm (n=40), and (3) poor image quality or artifacts (n=33). In total, the ICI group enrolled 111 patients with 151 GGNs. For patients with multiple GGNs, each GGN was assessed independently, while clinical data were shared at the patient level.

A comparator cohort was established from individuals with incidentally detected pulmonary nodules who underwent regular follow-up, and were identified through the institutional imaging system and health screening center database between June 2019 and September 2025. The inclusion criteria were: (1) exhibit GGNs on chest CT, (2) age > 40 years and< 80 years, and (3) availability of two chest CT scans ≥ 3 months apart. Initially, 673 patients with 673 GGNs were eligible. Exclusion criteria included: (1) prior anticancer therapy (n = 122); (2) baseline nodule diameter< 6 mm (n = 107); or (3) poor image quality or artifacts (n = 35). In total, the control cohort comprised 409 patients with 409 GGNs.

Propensity score matching (PSM) was performed at a 1:1 ratio based on age, sex, smoking history, baseline diameter, baseline volume and follow-up interval. After matching, 110 GGNs from 90 patients in the ICI group were matched to 110 GGNs from 110 patients in the control group. The remaining 41 GGNs in the ICI group were unmatched and therefore excluded from the matched analysis. Clinical data were collected for the ICI group, including age, gender, smoking history, histological subtype of the primary lung cancer, tumor size, location, and TNM stage (16).

### Treatment protocol

All patients were treated with 3–4 cycles of ICI therapy, consisting of sintilimab, camrelizumab, pembrolizumab, durvalumab, or tislelizumab according to the standard dosing regimen for each agent.

### CT acquisition

CT examinations were performed using multidetector spiral CT scanners (SOMATOM Definition, SOMATOM Definition AS+, Siemens Healthineers, Erlangen, Germany) without intravenous contrast. Scanning parameters were 64×0.6 mm or 128×0.6 mm collimation, 120 kV tube voltage, 1.0-1.5 mm slice thickness and increment. Reconstructed images were sent to the workstation and PACS for multiplanar reconstruction processing. Lung and mediastinal window were set at -600 HU/width 1200 HU and 50 HU/width 1200 HU, respectively.

### CT image interpretation

All imaging evaluations were independently performed by two thoracic radiologists (Reader 1 and Reader 2). The reviewers were blinded to clinical and pathological information while being informed of the nodule location. Discrepancies between the two radiologists were resolved through consensus discussion. Tumor size (calculated as the average the long- and short-axis diameters on axial CT images), density [pure ground-glass nodule (pGGN) or part-solid nodule (PSN)], margin (well defined or ill defined), spiculation, lobulation, vacuole sign, pleural indentation, and vascular convergence sign were assessed. All nodules were assessed using the Lung CT Screening Reporting and Data System (Lung-RADS) version 1.1 (17). Longitudinal changes in Lung-RADS categories were assessed and classified as improvement, stable, and progression.

### Quantitative parameters from follow-up CT imaging

The InferRead CT Lung system (version R13.3; Infervision Medical Technology Co., Ltd., Beijing, China) was used for automatically extracting quantitative image analysis (18–21). Automated segmentation results were reviewed by two thoracic radiologists (Reader 1 and Reader 2), and manual adjustments were applied when the automatically generated contours were inaccurate. Quantitative metrics were derived from baseline and follow-up CT images, including diameter (mm), volume (mm^3^), surface area (mm^2^), mass (mg), mean CT attenuation (HU), CT attenuation standard deviation (HU), percentage of solid component, sphericity as a shape feature, and energy and entropy as first-order radiomic features. Sphericity describes how closely a nodule approximates a sphere, whereas energy and entropy reflect the uniformity and heterogeneity of voxel intensity distribution, respectively (22). Solid and ground-glass components were defined using a -300 HU threshold. Given the variability in follow-up durations, quantitative parameters (X) were time-normalized using the Equations 1, 2

(1) $$Absolute\;monthly\;change\;of\;X=\left(X_{final}-X_{baseline}\right)/time\left(months\right)$$

(2) $$Relative\;monthly\;change\;of\;X=\left(X_{final}-X_{baseline}\right)/\left(X_{baseline}\times time\right)$$

Nodule response was defined by volume change proportion (VCP), calculated using Equation 3 with >25% increase as progression, >25% decrease as regression, and ±25% as stability (23, 24):

(3) $$VCP=\left(X_{final}-X_{baseline}\right)/\left(X_{baseline}\right)$$

### Statistical analysis

All statistical analyses were performed using R software (version 4.2.2; R Core Team, Vienna, Austria). PSM was performed to balance baseline characteristics between the two groups, and covariate balance after matching was assessed using standardized mean differences (SMDs), with an SMD< 0.10 generally considered indicative of adequate balance. Continuous variables were presented as medians with interquartile ranges (IQR), and categorical variables as counts with percentages. Normality was assessed using the Shapiro-Wilk test. Between-group comparisons were performed using the independent-samples t test for normally distributed continuous variables, the Mann-Whitney U test for non-normally distributed variables, and the chi-square test for categorical variables. Within-group comparisons employed the paired t test for normally distributed continuous variables, the Wilcoxon signed-rank test for non-normally distributed variables, and the McNemar test for categorical variables. Linear mixed-effects models were used to adjust for confounding variables (age, sex, smoking history, nodule location, and baseline size). Multiple comparisons were corrected using the Benjamini-Hochberg procedure for controlling false discovery rate. Subgroup analyses were conducted stratified by nodule density (pGGNs *vs*. PSNs) and baseline Lung-RADS category (2–3 *vs*. ≥ 4A).

## Results

### Baseline characteristics

After PSM, the ICI group included 90 patients (110 GGNs), and the control group included 110 patients (110 GGNs). Patients in both groups were predominantly male, accounting for 70.0% of the ICI group and 73.6% of the control group. The median age was 64 years in the ICI group and 63 years in the control group. Non-smokers comprised 65.5% and 55.5% of the ICI and control groups, respectively. pGGNs accounted for 48.2% in the ICI group and 42.7% in the control group. The median follow-up interval was 5.50 months in the ICI group and 4.00 months in the control group. Baseline characteristics were generally well balanced after matching. Although smoking history showed slight residual imbalance (SMD = 0.206), the between-group difference was not statistically significant (**Table 1**). Additional clinical characteristics of patients in the ICI group were summarized in **Supplementary Table 1**. The predominant primary tumor types were adenocarcinoma (57.2%) and squamous cell carcinoma (27.2%). Most patients (81.7%) were at stage III-IV and received ICI with a median of 4 treatment cycles.

**Table 1:** Characteristics of the ICI and control groups at the nodule level.

Variables	ICI group (n=110)	Controls (n=110)	p value
Gender			0.549
Female	33 (30.0%)	29 (26.3%)	
Male	77 (70.0%)	81 (73.6%)	
Age, years	64 [59, 69]	63 [58, 68]	0.563
Smoking history			0.129
No	72(65.5%)	61(55.5%)	
Yes	38 (34.5%)	49 (44.5%)	
Location			0.033*
RU	38 (34.5%)	52 (47.3%)	
RM	3 (2.7%)	8 (7.3%)	
RL	22 (20.0%)	11 (10.0%)	
LU	36 (32.7%)	25 (22.7%)	
LL	11 (10.0%)	14 (12.7%)	
Density			0.417
pGGN	53 (48.2%)	47 (42.7%)	
PSN	57 (51.8%)	63 (57.3%)	
Interval time, months	5.50 [3.03, 6.80]	4.00 [3.00, 7.00]	0.085
Baseline Lung-RADS			0.047*
2	54 (49.1%)	52 (47.3%)	
3	38 (34.5%)	24 (21.8%)	
4A	9 (8.2%)	19 (17.3%)	
4B	8 (7.3%)	10 (9.1%)	
4X	1 (0.9%)	5 (4.5%)	
Lung-RADs change			0.018*
Downgraded	9 (8.2%)	2 (1.8%)	
Unchanged	89 (80.9%)	85 (77.3%)	
Upgraded	12 (10.9%)	23 (20.9%)	
VCP			<0.001*
Regressive	26 (23.6%)	2 (1.8%)	
Stable	65 (59.1%)	87 (79.1%)	
Progressive	19 (17.3%)	21 (19.1%)	
X_Baseline			
Diameter, mm	9.1 [6.9, 12.7]	10.0 [7.5, 13.0]	0.169
Volume, mm3	251 [134, 1,113]	351 [201, 1,097]	0.067
Surface area, mm2	344 [211, 734]	412 [261, 809]	0.048*
Mass, mg	166 [73, 468]	230 [106, 508]	0.083
Mean CT value, Hu	-580 [-663, -467]	-558 [-640, -453]	0.169
Standard deviation	148 [113, 191]	158 [131, 192]	0.107
Solid component, %	0 [0, 0]	0 [0, 9]	0.005*
Sphericity	0.80 [0.75, 0.83]	0.79 [0.75, 0.83]	0.871
Energy	294,137,630 [134,211,979, 758,255,905]	356,873,175 [163,895,007, 1,068,498,389]	0.043*
Entropy	4.55 [4.21, 4.93]	4.72 [4.42, 5.04]	0.028*
ΔX_monthly			
Diameter	0.00 [-0.23, 0.00]	0.00 [0.00, 0.12]	<0.001*
Volume	-2 [-25, 7]	7 [-5, 26]	<0.001*
Surface area	-1 [-12, 8]	5 [-4, 22]	0.005*
Mass	0 [-7, 8]	2 [-1, 12]	0.035*
Mean CT value	4 [-6, 15]	0 [-4, 10]	0.088
Standard deviation	2 [-3, 6]	-1 [-6, 3]	0.006*
Solid component	0.00 [-0.01, 0.02]	0.00 [-0.02, 0.03]	0.457
Sphericity	-0.001 [-0.007, 0.004]	0.000 [-0.006, 0.003]	0.785
Energy	-3,763,679 [-41,863,835, 15,992,385]	-8,873,579 [-51,673,199, 13,102,635]	0.407
Entropy	0.01 [-0.04, 0.08]	-0.01 [-0.05, 0.03]	0.011*
X_change_monthly			
Diameter	-0.1 [-2.4, 0.0]	0.0 [0.0, 1.1]	<0.001*
Volume	-1 [-4, 3]	2 [-2, 6]	0.001*
Surface area	0 [-2, 2]	1 [-1, 5]	0.007*
Mass	0 [-3, 3]	2 [-1, 4]	0.035*
Mean CT value	-0.7 [-3.5, 1.0]	0.0 [-1.9, 1.0]	0.057
Standard deviation	1 [-2, 5]	-1 [-4, 3]	0.006*
Solid component, %	5 [-5, 27]	0 [-5, 12]	0.206
Sphericity	-0.15 [-0.96, 0.48]	-0.06 [-0.75, 0.40]	0.794
Energy	-3 [-9, 6]	-3 [-11, 6]	0.817
Entropy	0.30 [-0.77, 1.64]	-0.21 [-1.09, 0.75]	0.013*

Data are n (%), n/N (%), or median (IQR). IQR, interquartile range. X_Baseline represents the baseline value of parameter X at the initial CT scan. ΔX denotes the absolute change of parameter X between two time points. ΔX_monthly indicates the absolute rate of change per month, calculated as ΔX divided by follow-up interval time (months). X_change_monthly refers to the relative percentage rate of change per month, calculated as (ΔX/X_B) × 100% divided by follow-up interval time (months).

ICI, Immune checkpoint inhibitor; RU, right upper lobe; RM, right middle lobe; RL, right lower lobe; LU, left upper lobe; LL, left lower lobe; pGGN, pure ground-glass nodule; PSN, part-solid nodule; VCP, volume change proportion.

*, p< 0.05 was considered statistically significant.

### GGNs evolution between groups

**Figure 2:**
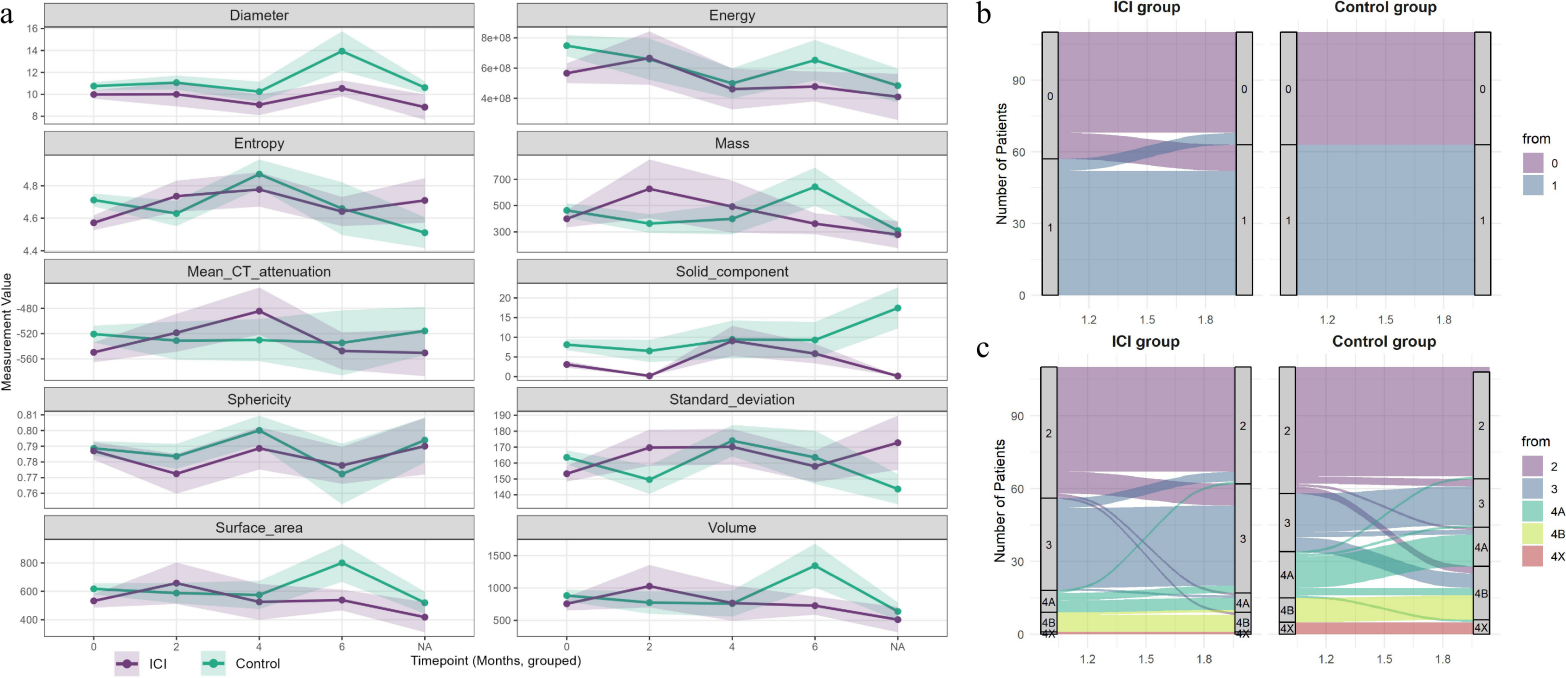
Overall trends of imaging quantitative parameter changes over 6 months in the ICI and control groups **(a)**. The changes in GGNs density **(b)** and Lung-RADS scores **(c)** between the ICI and control groups.

**Figure 3:**
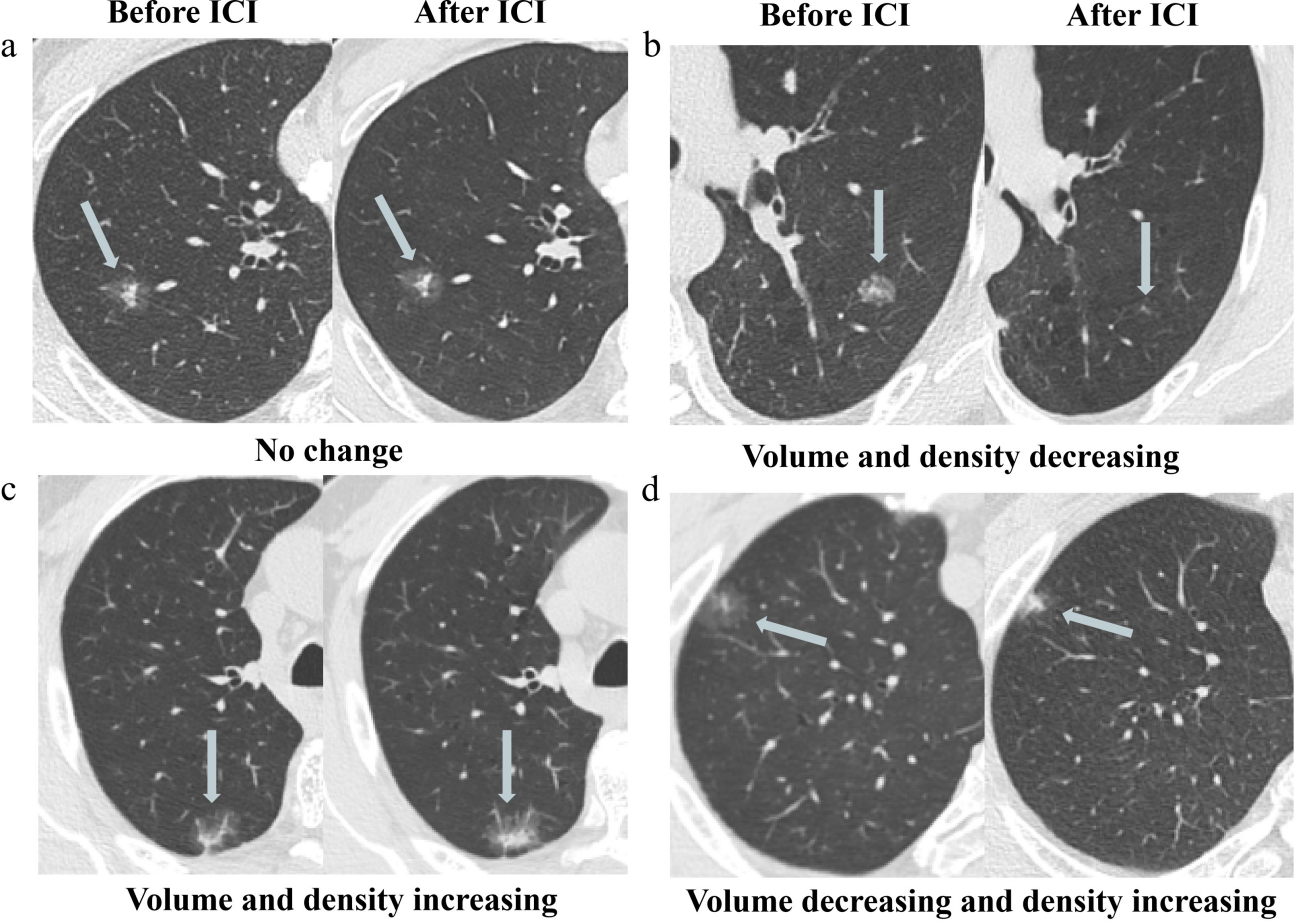
Changes in four representative cases during follow up in the ICI group. **(a)** sustained stability with no appreciable change; **(b)** gradual decreases in both size and density; **(c)** progressive increases in volume and density; and **(d)** decreasing volume accompanied by increasing density.

Based on a ± 25% volumetric threshold, nodules in ICI group were more likely to show regression than those in the control group (23.6% *vs*. 1.8%, p< 0.001). Lung-RADS shifts mirrored these trends. In the ICI group, 8.2% of nodules were downgraded, 80.9% remained unchanged, and 10.9% were upgraded, compared with 1.8%, 77.3%, and 20.9%, respectively, in the control group (p = 0.018) (**Table 1**; **Figure 2**). During follow up, GGNs in the ICI group exhibited four distinct evolutionary trajectories: (1) sustained stability with no appreciable change; (2) gradual decreases in both size and density; (3) progressive increases in volume and density; and (4) decreasing volume accompanied by increasing density (**Figure 3**).

### Between-group differences in longitudinal CT changes

**Figure 4:**
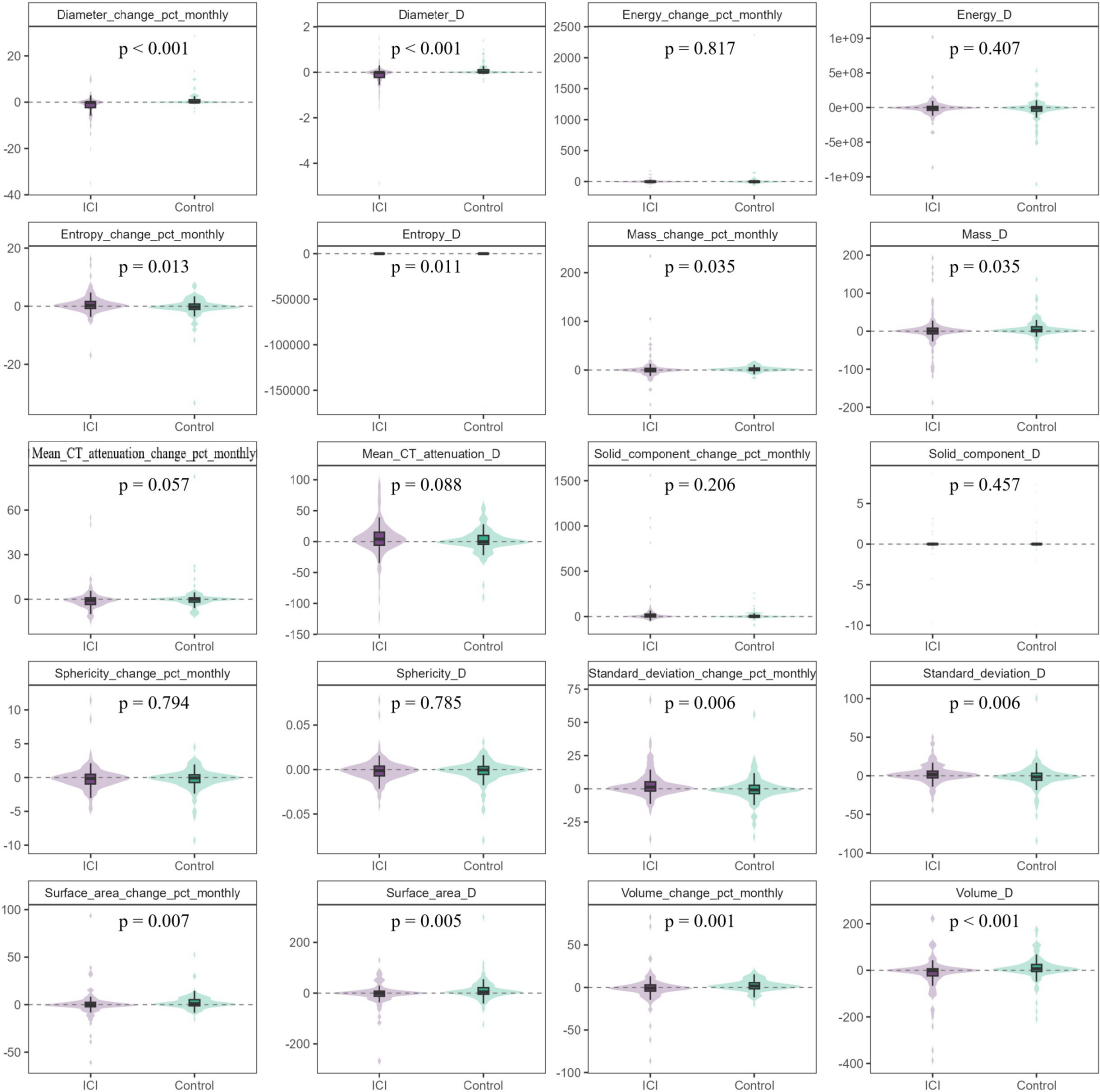
Comparison of absolute and relative monthly changes in quantitative imaging parameters between the ICI and control groups. X_D indicates the absolute monthly change, and X_change_pct_monthly refers to the relative monthly change. Quantitative parameters included diameter, energy, entropy, mass, mean CT attenuation, solid component, sphericity, CT attenuation standard deviation, surface area, and volume.

During follow-up, GGNs in the ICI group exhibited significantly smaller monthly changes in diameter (0 [-0.23, 0.00] mm/month *vs*. 0 [0.00, 0.12] mm/month, p< 0.001), volume (-2 mm^3^/month *vs*. 7 mm^3^/month, p< 0.001), surface area (-1 mm^2^/month *vs*. 5 mm^2^/month, p = 0.005), and mass (0 mg/month *vs*. 2 mg/month, p = 0.035) than those in the control group. In contrast, the ICI group demonstrated significantly higher absolute monthly increases in standard deviation (2 Hu/month *vs*.-1 Hu/month, p = 0.006) and entropy (0.01/month *vs*. -0.01/month, p = 0.011). Relative change analyses showed consistent findings for diameter (-0.1%/month *vs*. 0%/month, p< 0.001), volume (-1%/month *vs*. 2%/month, p = 0.001), surface area (0%/month *vs*. 1%/month, p = 0.007), mass (0%/month *vs*. 2%/month, p = 0.035), standard deviation (1%/month *vs*. -1%/month, p = 0.006), and entropy (0.30%/month *vs*. -0.21%/month, p = 0.013) (**Table 1**; **Figure 4**). After adjustment for potential confounders, nodules in the ICI group showed a 0.254 mm/month lower change in diameter (95% CI: -0.384 to -0.124, p< 0.001), a 26.693 mm^3^/month lower change in volume (95% CI: -45.602 to -7.784, p = 0.039) and a 2.671%/month greater relative decrease in diameter (95% CI: -3.954 to -1.389, p< 0.001) than those in the control group (**Supplementary Table 2**). No other parameters remained statistically significant after adjustment. Exploratory analyses revealed that larger baseline nodules tended to grow more rapidly, while this association was diminished among patients receiving ICI therapy (**Supplementary Figure 1**).

### Within-group longitudinal changes from baseline to follow-up

**Figure 5:**
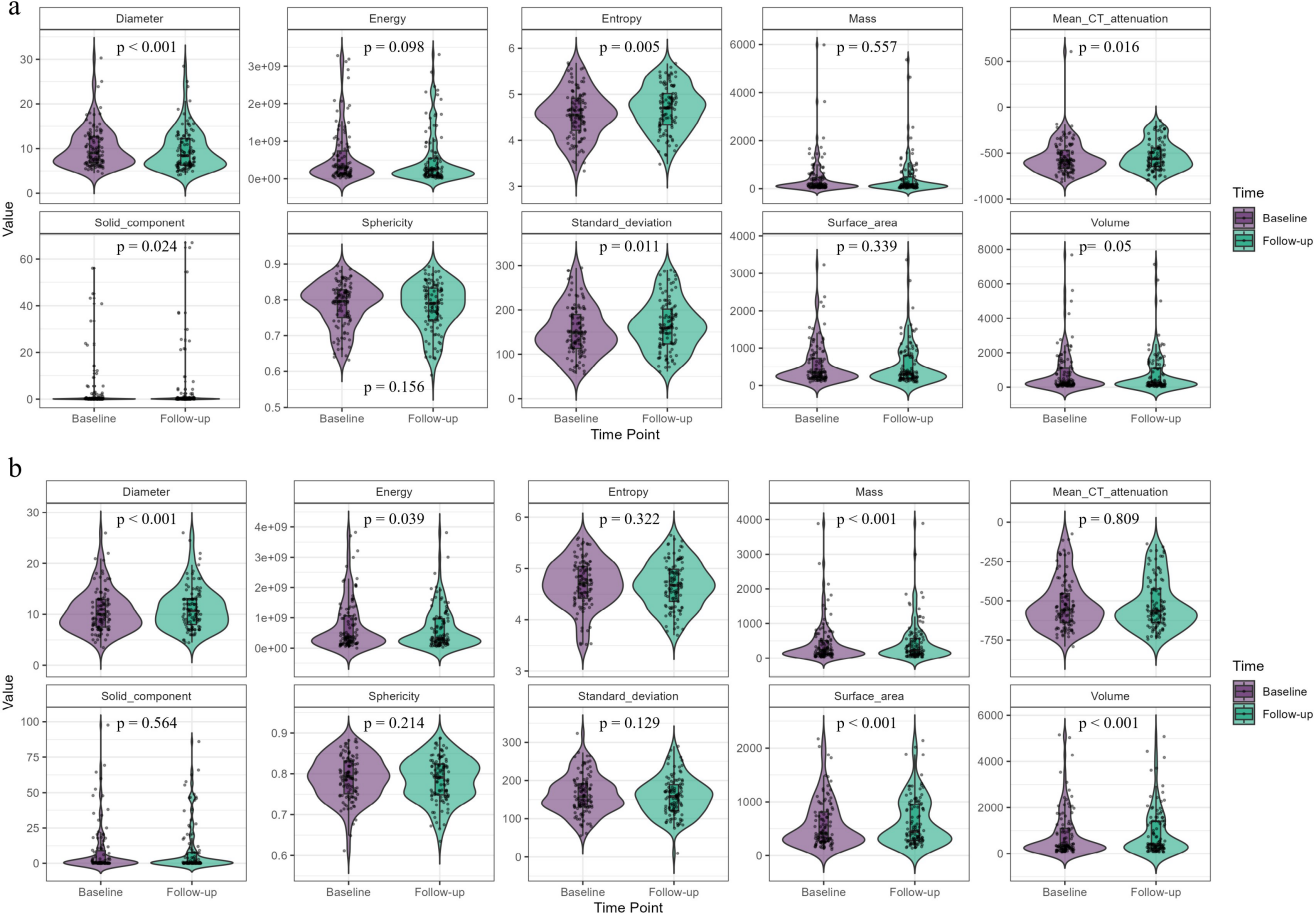
Comparison of baseline and follow-up quantitative imaging parameters for the ICI **(a)** and control groups **(b)**. Quantitative parameters included diameter, energy, entropy, mass, mean CT attenuation, solid component, sphericity, CT attenuation standard deviation, surface area, and volume.

In the ICI group, quantitative analysis showed a significant reduction in nodule diameter (p< 0.001), with significant increases in mean CT value (p = 0.016), standard deviation (p = 0.011), solid component proportion (p = 0.024), and entropy (p = 0.005) during following-up. Conversely, nodules in the control group significantly enlarged in diameter, volume, surface area, mass (all p<0.001), with a decrease in energy (p = 0.039) (**Table 2**; **Figure 5**). No significant changes in morphological features were observed in either groups (**Table 2**).

**Table 2:** Comparison of quantitative characteristics at baseline and follow-up in the ICI and control groups.

Variables	ICI group		p value	Controls		p value
	Baseline	Follow-up		Baseline	Follow-up	
Diameter, mm	9.05 [6.86, 12.64]	8.43 [6.30, 12.24]	<0.001*	10.00 [7.50, 12.97]	10.70 [8.00, 13.07]	<0.001*
Volume, mm3	251.00 [134.00, 1104.25]	230.00 [116.00, 1095.25]	0.050	350.50 [202.50, 1094.00]	402.50 [223.50, 1406.25]	<0.001*
Surface area, mm2	344.00 [211.25, 728.75]	304.50 [192.00, 803.75]	0.339	412.00 [263.50, 807.75]	450.50 [281.75, 951.75]	<0.001*
Mass, mg	165.70 [74.01, 458.89]	155.72 [71.54, 488.04]	0.557	229.83 [106.21, 506.93]	229.03 [110.72, 568.72]	<0.001*
Mean CT value, Hu	-580.00 [-662.50, -469.50]	-560.50 [-644.50, -444.25]	0.016*	-558.00 [-639.50, -454.00]	-565.50 [-639.00, -423.75]	0.809
Standard deviation	148.03 [113.10, 189.77]	159.18 [123.24, 201.89]	0.011*	157.83 [130.70, 191.55]	158.44 [121.28, 189.22]	0.129
Solid component, %	0.13 [0.03, 0.46]	0.18 [0.04, 0.58]	0.024*	0.38 [0.04, 9.06]	0.37 [0.04, 7.77]	0.564
Sphericity	0.80 [0.75, 0.83]	0.79 [0.74, 0.83]	0.156	0.79 [0.75, 0.83]	0.79 [0.75, 0.82]	0.214
Energy	294137629.50 [134961577.00, 741852803.25]	237774144.50 [92534554.50, 549853315.00]	0.098	356873174.50 [166220411.50, 1062223556.00]	322379929.00 [177047340.25, 964134522.25]	0.039*
Entropy	4.55 [4.22, 4.91]	4.71 [4.34, 5.01]	0.005*	4.72 [4.42, 5.04]	4.67 [4.36, 4.98]	0.322
Well-defined margin	34 (30.9)	31 (28.2)	0.546	44 (40)	49 (44.5)	0.074
Spiculation	9 (8.2)	9 (8.2)	1	10 (9.1)	10 (9.1)	1
Lobulation	14 (12.7)	13 (11.8)	1	15 (13.6)	17 (15.5)	1
Vacuole	22 (20.0)	22 (20.0)	1	17 (15.5)	17 (15.5)	1
Pleural indentation	27 (24.5)	28 (25.5)	1	32 (29.1)	32 (29.1)	1
Vascular convergence	70 (63.6)	72 (65.5)	0.617	75 (68.2)	75 (68.2)	1

Data are n (%), n/N (%), or median (IQR). IQR, interquartile range.

*p< 0.05 was considered statistically significant.

### Subgroup analyses

As shown in **Supplementary Tables 3**, **4**, most nodules remained stable on Lung-RADS assessment across subgroups. Upgrading was more frequent in pGGNs than in PSNs (18.9% *vs*. 3.5%, p = 0.001). Nodules with baseline Lung-RADS ≥ 4A were more likely to be downgraded than those with baseline Lung-RADS< 4A (27.8% *vs*. 4.3%, p = 0.002). PSNs showed more decreases in volume than pGGNs (p = 0.006). Nodules with baseline Lung-RADS ≥ 4A also showed a significant decrease in entropy (p = 0.032). **Supplementary Figure 2** showed trends of quantitative parameters across subgroups.

### Pathological validation

In the ICI group, 5 nodules were pathologically confirmed, including one minimally invasive adenocarcinoma (MIA) and four invasive adenocarcinomas (IACs). In the control group, pathological confirmation was available for 11 of the 110 incidentally detected pulmonary nodules, including 4 MIAs and 7 IACs. Among histologically confirmed GGNs, the ICI group showed greater absolute and relative monthly increases in CT attenuation standard deviation, as well as greater relative monthly increases in solid component, compared to the control group (all p< 0.05). Given the limited number of pathologically confirmed nodules, these findings should be considered exploratory. A comparison of histologically confirmed GGNs between the ICI and control groups is shown in **Supplementary Table 5**.

## Discussion

In this study, quantitative CT analysis showed that synchronous GGNs in lung cancer patients in the ICI group exhibited slower longitudinal growth than matched controls. Although most nodules remained radiologically stable according to conventional volumetric and Lung-RADS criteria, subtle yet consistent differences in growth kinetics were detectable using quantitative imaging metrics, particularly in PSNs and lesions with higher baseline Lung-RADS categories. These findings support the utility of CT-based quantitative analysis in capturing subclinical imaging changes in indolent pulmonary nodules during follow up, rather than indicating direct therapeutic efficacy.

Recent studies have investigated the impact of immunotherapy on GGNs. Wu et al. (25) reported that in a patient with more than 30 GGNs treated with ICIs, nearly all nodules achieved radiographic complete remission during 1 year of follow-up. Cheng et al. (13) further showed that PD-1 inhibitor exhibited certain activity on high-risk pulmonary GGNs, which was associated with immune modulation and metabolism pathways regulation. However, in advanced lung adenocarcinoma with synchronous GGNs, ICIs achieved an objective response rate of 55.6% in primary tumors, whereas only 8.1% in GGNs (26). A single-arm phase II trial also reported that none of the GGNs treated with tislelizumab met the criteria for complete or partial response (27). Nevertheless, these negative results were insufficient to conclude that GGNs are biologically unresponsive. Xu et al. (15) enrolled 21 patients with multiple primary lung cancers treated with sintilimab and found that, although nodule size remained largely unchanged, 2 of 13 resected GGNs achieved major pathological response after immunotherapy. These findings suggests that conventional size-based criteria alone may be insufficient to capture short-term changes in these indolent lesions.

In our study, nodule diameter decreased significantly during follow-up in the ICI group, while mean CT value, standard deviation, solid component and entropy increased significantly. This pattern suggests that, despite an apparent reduction in nodule size, internal heterogeneity may have increased over time. A similar trend was also observed in the exploratory analysis of histologically confirmed GGNs, in which the monthly increases in standard deviation and solid component were greater in the ICI group than in the control group. Such changes may reflect immune cell infiltration, inflammatory response, or fibrosis during treatment (8, 28). Nevertheless, given the limited pathological validation, these interpretations remain speculative. Larger studies are warranted to validate these findings and to further clarify the underlying molecular mechanisms.

Subgroup analyses further suggested that imaging evolution patterns may vary according to baseline nodule characteristics and risk stratification. Lung-RADS category downgrading during follow-up was more frequently observed in PSNs and in nodules with a baseline Lung-RADS ≥ 4A. Moreover, PSNs showed a greater reduction in volume than pGGNs, suggesting that nodules with a larger solid component may be more likely to exhibit measurable short-term CT changes. Notably, this subgroup-specific pattern differed from the overall increase in entropy observed in the entire ICI cohort. Entropy significantly decreased in nodules with a baseline Lung-RADS ≥ 4A, which may indicate reduced internal heterogeneity in this higher-risk subgroup. Although entropy may help capture subtle internal changes in GGNs that are not readily appreciable on conventional assessment, its direct role in clinical decision-making remains uncertain and warrants further investigation.

Genomic analyses reported that GGNs exhibit lower copy-number variants (CNV) burden than invasive adenocarcinoma, which may reduce tumor-associated antigen availability and thereby limit immune recognition (29). Transcriptomic analyses showed that, compared with solid nodules, GGNs harbor a relatively less active immune microenvironment with reduced activity of immune-related pathways (8). T-cell repertoire sequencing further indicated reduced T-cell expansion in GGNs (8). Clinical evidence has demonstrated that higher T-cell receptor clonality was strongly correlated with enhanced antitumor immunity and superior therapeutic outcomes (30). With lesion progression, lymphocytes are seen to infiltrate and there is increased CD4 infiltration with reduced CD8 cell infiltration leading to an increased CD4:CD8 ratio (31, 32). These findings provide a potential explanation for the more pronounced favorable short-term CT changes observed in PSNs and Lung-RADS≥4A lesions in our study.

Considering the natural course of ground-glass nodules and their temporal changes on CT imaging, we included an incidental nodule control group to reduce potential confounding factor. Because this control group consisted of individuals with incidentally detected pulmonary nodules rather than patients with lung cancer, tumor-related clinical information, such as tumor stage, histologic subtype, and tumor burden, was not available for this group. Accordingly, the present comparison should be interpreted as comparative descriptive evidence rather than direct evidence of an ICI-specific influence on synchronous GGNs. Future prospective studies incorporating a more appropriate control cohort, such as patients with advanced lung cancer and synchronous GGNs who do not receive ICIs, are still needed to further clarify the impact of ICI therapy on synchronous GGNs.

The study had some limitations. First, as a retrospective study, potential selection bias and residual confounding cannot be fully excluded, although the major matching covariates were generally balanced after propensity score matching. Second, only a small proportion of nodules in both groups had histopathological confirmation, limiting the biological interpretation of the imaging findings. Third, the relatively short follow-up duration may not fully capture the true biological evolution of indolent GGNs and limits the assessment of long-term outcomes. Finally, automated quantitative analysis was not independently validated in our cohort, and potentially relevant factors, including treatment-line heterogeneity, driver mutations, and PD-L1 expression, were not fully accounted for.

In summary, among lung cancer patients receiving ICIs, synchronous GGNs exhibited attenuated longitudinal CT growth patterns, with favorable shifts in VCP and Lung-RADS categories, particularly in PSNs and nodules with baseline Lung-RADS ≥ 4A. These findings support the utility of quantitative imaging metrics for capturing subtle longitudinal changes. Further studies with larger cohorts, longer follow-up, and multimodal biomarker integration are warranted.

## Data Availability

The raw data supporting the conclusions of this article will be made available by the authors, without undue reservation.

## References

[1] Wu Y He S Cao M Teng Y Li Q Tan N . Comparative analysis of cancer statistics in China and the United States in 2024. Chin Med J (Engl). (2024) 137:3093–100. doi: 10.1097/CM9.0000000000003442 39654104 PMC11706596

[2] de Koning HJ van der Aalst CM de Jong PA Scholten ET Nackaerts K Heuvelmans MA . Reduced lung-cancer mortality with volume CT screening in a randomized trial. N Engl J Med. (2020) 382:503–13. doi: 10.1056/NEJMoa1911793 31995683

[3] Chang B Hwang JH Choi YH Chung MP Kim H Kwon OJ . Natural history of pure ground-glass opacity lung nodules detected by low-dose CT scan. Chest. (2013) 143:172–8. doi: 10.1378/chest.11-2501 22797081

[4] Hillerdal G . Indolent lung cancers--time for a paradigm shift: a review. J Thorac Oncol. (2008) 3:208–11. doi: 10.1097/JTO.0b013e3181653ce3 18317061

[5] Yang H Sun Y Yao F Yu K Gu H Han B . Surgical therapy for bilateral multiple primary lung cancer. Ann Thorac Surg. (2016) 101:1145–52. doi: 10.1016/j.athoracsur.2015.09.028 26602007

[6] Borghaei H Gettinger S Vokes EE Chow LQM Burgio MA de Castro Carpeño J . Five-year outcomes from the randomized, phase III trials CheckMate 017 and 057: Nivolumab versus docetaxel in previously treated non-small-cell lung cancer. J Clin Oncol. (2021) 39:723–33. doi: 10.1200/JCO.20.01605 33449799 PMC8078445

[7] Mountzios G Remon J Hendriks LEL García-Campelo R Rolfo C Van Schil P . Immune-checkpoint inhibition for resectable non-small-cell lung cancer - opportunities and challenges. Nat Rev Clin Oncol. (2023) 20:664–77. doi: 10.1038/s41571-023-00794-7 37488229

[8] Chen K Bai J Reuben A Zhao H Kang G Zhang C . Multiomics analysis reveals distinct immunogenomic features of lung cancer with ground-glass opacity. Am J Respir Crit Care Med. (2021) 204:1180–92. doi: 10.1164/rccm.202101-0119OC 34473939 PMC8759311

[9] Qu R Ye F Hu S Wang B Qin S Xiong J . Distinct cellular immune profiles in lung adenocarcinoma manifesting as pure ground glass opacity versus solid nodules. J Cancer Res Clin Oncol. (2023) 149:3775–88. doi: 10.1007/s00432-022-04289-3 35986758 PMC11797326

[10] Dejima H Hu X Chen R Zhang J Fujimoto J Parra ER . Immune evolution from preneoplasia to invasive lung adenocarcinomas and underlying molecular features. Nat Commun. (2021) 12:2722. doi: 10.1038/s41467-021-22890-x 33976164 PMC8113327

[11] Mascaux C Angelova M Vasaturo A Beane J Hijazi K Anthoine G . Immune evasion before tumour invasion in early lung squamous carcinogenesis. Nature. (2019) 571:570–5. doi: 10.1038/s41586-019-1330-0 31243362

[12] Huang Y Kim BYS Chan CK Hahn SM Weissman IL Jiang W . Improving immune-vascular crosstalk for cancer immunotherapy. Nat Rev Immunol. (2018) 18:195–203. doi: 10.1038/nri.2017.145 29332937 PMC5922422

[13] Cheng B Li C Li J Gong L Liang P Chen Y . The activity and immune dynamics of PD-1 inhibition on high-risk pulmonary ground glass opacity lesions: insights from a single-arm, phase II trial. Signal Transduct Target Ther. (2024) 9:93. doi: 10.1038/s41392-024-01799-z 38637495 PMC11026465

[14] Park MD Hu X Montégut L Zhu B Vokes N Fujimoto J . PP01.23/OA07.01: Myeloid IL-1 signaling drives the precancer-to-cancer evolution of human pulmonary nodules. J Thorac Oncol. (2026) 21:S45–6. doi: 10.1016/j.jtho.2025.12.098 38826717

[15] Xu L Shi M Wang S Li M Yin W Zhang J . Immunotherapy for bilateral multiple ground glass opacities: An exploratory study for synchronous multiple primary lung cancer. Front Immunol. (2022) 13:1009621. doi: 10.3389/fimmu.2022.1009621 36389707 PMC9642914

[16] Rami-Porta R Nishimura KK Giroux DJ Detterbeck F Cardillo G Edwards JG . The International Association for the Study of Lung Cancer Lung Cancer Staging Project: Proposals for revision of the TNM stage groups in the forthcoming (ninth) edition of the TNM classification for lung cancer. J Thorac Oncol. (2024) 19:1007–27. doi: 10.1016/j.jtho.2024.02.011 38447919

[17] Christensen J Prosper AE Wu CC Chung J Lee E Elicker B . ACR Lung-RADS v2022: Assessment categories and management recommendations. Chest. (2024) 165:738–53. doi: 10.1016/j.chest.2023.10.028 38300206

[18] Pei C Wu F Yang M Pan L Ding W Dong J . Multi-source domain adaptation for medical image segmentation. IEEE Trans Med Imaging. (2024) 43:1640–51. doi: 10.1109/TMI.2023.3346285 38133966

[19] Chen X Dai C Peng M Wang D Sui X Duan L . Artificial intelligence driven 3D reconstruction for enhanced lung surgery planning. Nat Commun. (2025) 16:4086. doi: 10.1038/s41467-025-59200-8 40312393 PMC12046031

[20] Liu K Li Q Ma J Zhou Z Sun M Deng Y . Evaluating a fully automated pulmonary nodule detection approach and its impact on radiologist performance. Radiol Artif Intell. (2019) 1:e180084. doi: 10.1148/ryai.2019180084 33937792 PMC8017422

[21] Wang Y Yan F Lu X Zheng G Zhang X Wang C . IILS: Intelligent imaging layout system for automatic imaging report standardization and intra-interdisciplinary clinical workflow optimization. EBioMedicine. (2019) 44:162–81. doi: 10.1016/j.ebiom.2019.05.040 31129095 PMC6604879

[22] The image biomarker standardization initiative: standardized quantitative radiomics for high-throughput image-based phenotyping. PubMed (Accessed April 19, 2026). 10.1148/radiol.2020191145 PMC7193906 32154773

[23] Callister MEJ Baldwin DR Akram AR Barnard S Cane P Draffan J . British Thoracic Society guidelines for the investigation and management of pulmonary nodules. Thorax. (2015) 70:ii1–ii54. doi: 10.1136/thoraxjnl-2015-207168 26082159

[24] Horeweg N van der Aalst CM Vliegenthart R Zhao Y Xie X Scholten ET . Volumetric computed tomography screening for lung cancer: three rounds of the NELSON trial. Eur Respir J. (2013) 42:1659–67. doi: 10.1183/09031936.00197712 23845716

[25] Wu S Li D Chen J Chen W Ren F . Tailing effect of PD-1 antibody results in the eradication of unresectable multiple primary lung cancer presenting as ground-glass opacities: a case report. Ann Palliat Med. (2021) 10:778–84. doi: 10.21037/apm-20-2132 33545799

[26] Wu F Li W Zhao W Zhou F Xie H Shi J . Synchronous ground‐glass nodules showed limited response to anti‐PD‐1/PD‐L1 therapy in patients with advanced lung adenocarcinoma. Clin Transl Med. (2020) 10:e149. doi: 10.1002/ctm2.149 41531421

[27] Wu J Fu X Gao Y Qin S Xiong J Qu R . The impact of tislelizumab immunotherapy on multiple primary lung cancer presenting as ground-glass nodules: preliminary results analysis from a single-arm, phase II trial. Cancer Immunol Immunother CII. (2025) 74:353. doi: 10.1007/s00262-025-04212-y 41182404 PMC12583299

[28] Li Y Li X Chen H Sun K Li H Zhou Y . Single-cell RNA sequencing reveals the multi-cellular ecosystem in different radiological components of pulmonary part-solid nodules. Clin Transl Med. (2022) 12:e723. doi: 10.1002/ctm2.723 35184398 PMC8858630

[29] Wu N Liu S Li J Hu Z Yan S Duan H . Deep sequencing reveals the genomic characteristics of lung adenocarcinoma presenting as ground-glass nodules (GGNs). Transl Lung Cancer Res. (2021) 10:1239–55. doi: 10.21037/tlcr-20-1086 33889506 PMC8044491

[30] Zhang J Ji Z Caushi JX El Asmar M Anagnostou V Cottrell TR . Compartmental analysis of T-cell clonal dynamics as a function of pathologic response to neoadjuvant PD-1 blockade in resectable non-small cell lung cancer. Clin Cancer Res. (2020) 26:1327–37. doi: 10.1158/1078-0432.CCR-19-2931 31754049 PMC7073288

[31] Succony L Rassl DM Barker AP McCaughan FM Rintoul RC . Adenocarcinoma spectrum lesions of the lung: Detection, pathology and treatment strategies. Cancer Treat Rev. (2021) 99:102237. doi: 10.1016/j.ctrv.2021.102237 34182217

[32] Yanagawa J Tran LM Salehi-Rad R Lim RJ Dumitras C Fung E . Single-cell characterization of pulmonary nodules implicates suppression of immunosurveillance across early stages of lung adenocarcinoma. Cancer Res. (2023) 83:3305–19. doi: 10.1158/0008-5472.CAN-23-0128 37477508 PMC10544016

