# Supplementary material for: Short-term quantitative CT changes in synchronous ground-glass nodules during immune checkpoint inhibitor therapy in patients with lung cancer
Source: Front Immunol. 2026 Jun 24;17:1810440. doi: 10.3389/fimmu.2026.1810440 (PMC13341620; doi:10.3389/fimmu.2026.1810440)
Supplement: Supplementary file 1 [file Table1.docx]

| Table S1 Clinicopathological characteristics of the ICI group | |
| --- | --- |
|  | ICI group (n=110) |
| Primary tumor |  |
| Adenocarcinoma | 63 (57.2%) |
| Squamous cell carcinoma | 30 (27.2%) |
| Other | 17 (15.4%) |
| Primary tumor loaction |  |
| Central | 36 (32.7%) |
| Peripheral | 74 (67.2%) |
| TNM stage |  |
| I-II | 20(18.1%) |
| III | 31(28.1%) |
| IV | 59(53.6%) |
| Data are n (%), n/N (%), or median (IQR). SCLC, Others in lung cancers include small cell lung cancer, adenosquamous carcinoma and sarcomatoid carcinoma. TNM stage was defined according to the 9th edition of the AJCC Tumor-Node-Metastasis classification. | |

| Table S2 Mixed-effects model results for patients group effect on parameter changes | | | | | |
| --- | --- | --- | --- | --- | --- |
| Outcome | Estimate | CI Lower | CI Upper | p-value | p_adj |
| **ΔX_monthly** |  |  |  |  |  |
| Diameter | -0.254 | -0.384 | -0.124 | <0.001* | 0.002 * |
| Volume | -26.693 | -45.602 | -7.784 | 0.006* | 0.039 * |
| Surface area | -15.103 | -27.811 | -2.394 | 0.02* | 0.096 |
| Mass | -6.952 | -17.398 | 3.495 | 0.191 | 0.318 |
| Mean CT value | 3.257 | -3.833 | 10.346 | 0.366 | 0.488 |
| Standard deviation | 4.394 | 0.162 | 8.625 | 0.042* | 0.106 |
| Solid component | -0.17 | -0.616 | 0.276 | 0.453 | 0.532 |
| Sphericity | 0.001 | -0.002 | 0.005 | 0.453 | 0.532 |
| Energy | 28118484.14 | -17679937.7 | 73916905.97 | 0.228 | 0.351 |
| Entropy | 1688.533 | -1459.294 | 4836.359 | 0.292 | 0.417 |
| Δ**X_change_monthly**X_change_pct_monthly |  |  |  |  |  |
| Diameter | -2.671 | -3.954 | -1.389 | <0.001* | 0.001 * |
| Volume | -2.818 | -6.518 | 0.882 | 0.135 | 0.270 |
| Surface area | -2.178 | -5.346 | 0.99 | 0.177 | 0.318 |
| Mass | 1.832 | -3.827 | 7.491 | 0.524 | 0.551 |
| Mean CT attenuation | -0.425 | -2.858 | 2.008 | 0.731 | 0.731 |
| Standard deviation | 3.422 | 0.457 | 6.386 | 0.024* | 0.096 |
| Solid component | 46.664 | 1.6 | 91.728 | 0.043* | 0.106 |
| Sphericity | 0.177 | -0.324 | 0.678 | 0.487 | 0.541 |
| Energy | -34.837 | -79.141 | 9.467 | 0.123 | 0.270 |
| Entropy | 1.183 | 0.117 | 2.249 | 0.03* | 0.099 |
| CI denotes the 95% confidence interval, p-values are from the mixed-effects model, p_adj represents adjusted p-values using the false discovery rate (FDR) method. ΔX denotes the absolute change of parameter X between two time points. ΔX_monthly indicates the absolute rate of change per month, calculated as ΔX divided by follow-up interval time (months). ΔX_change_monthly refers to the relative percentage rate of change per month, calculated as (ΔX / X_B) × 100% divided by follow-up interval time (months).  *, p<0.05 was considered statistically significant. | | | | | |

| Table S3 Characteristics in density subgroups between the ICI and control groups. | | | | | | |
| --- | --- | --- | --- | --- | --- | --- |
|  | ICI group | |  | Controls | |  |
|  | pGGN (n=53) | PSN (n=57) | p value | pGGN (n=47) | PSN (n=63) | p value |
| Gender |  |  | 0.560 |  |  | 0.962 |
| Female | 14 (26.4) | 19 (33.3) |  | 13 (27.7) | 16 (25.4) |  |
| Male | 39 (73.6) | 38 (66.7) |  | 34 (72.3) | 47 (74.6) |  |
| Age, years | 63.00 [59.00, 69.00] | 64.00 [60.00, 68.00] | 0.581 | 64.00 [58.50, 68.50] | 62.00 [57.50, 68.00] | 0.797 |
| Smoking History |  |  | 0.054 |  |  | 0.085 |
| No | 40 (75.5) | 32 (56.1) |  | 31 (66.0) | 30 (47.6) |  |
| Yes | 13 (24.5) | 25 (43.9) |  | 16 (34.0) | 33 (52.4) |  |
| Location |  |  | 0.296 |  |  | 0.150 |
| RU | 16 (30.2) | 22 (38.6) |  | 28 (59.6) | 24 (38.1) |  |
| RM | 3 (5.7) | 0 (0.0) |  | 4 (8.5) | 4 (6.3) |  |
| RL | 10 (18.9) | 12 (21.1) |  | 4 (8.5) | 7 (11.1) |  |
| LU | 17 (32.1) | 19 (33.3) |  | 8 (17.0) | 17 (27.0) |  |
| LL | 7 (13.2) | 4 (7.0) |  | 3 (6.4) | 11 (17.5) |  |
| Interval time,months | 4.23 [2.87, 6.63] | 5.80 [3.27, 6.87] | 0.351 | 4.00 [2.50, 7.00] | 4.00 [2.00, 7.00] | 0.860 |
| Lung RADs |  |  | 0.001* |  |  | 0.008* |
| Downgraded | 0 (0.0) | 9 (15.8) |  | 0 (0.0) | 2 (3.2) |  |
| Unchanged | 43 (81.1) | 46 (80.7) |  | 43 (91.5) | 42 (66.7) |  |
| Upgraded | 10 (18.9) | 2 (3.5) |  | 4 (8.5) | 19 (30.2) |  |
| VCP |  |  | 0.05 |  |  | 0.186 |
| Progressive | 11 (20.8) | 15 (26.3) |  | 0 (0.0) | 2 (3.2) | 0.467 |
| Regressive | 28 (52.8) | 37 (64.9) |  | 38 (80.9) | 49 (77.8) |  |
| Stable | 14 (26.4) | 5 (8.8) |  | 9 (19.1) | 12 (19.0) |  |
| **X_Baseline** |  |  |  |  |  |  |
| Diameter, mm | 7.55 [6.35, 10.25] | 10.50 [7.65, 14.05] | 0.001* | 8.50 [7.00, 10.00] | 11.70 [8.80, 14.55] | <0.001* |
| Volume, mm^3^ | 172.00 [122.00, 337.00] | 745.00 [182.00, 1807.00] | <0.001* | 224.00 [134.00, 389.50] | 689.00 [315.50, 1759.00] | <0.001* |
| Surface area, mm^2^ | 255.00 [189.00, 355.00] | 612.00 [230.00, 1079.00] | 0.001* | 295.00 [211.00, 490.50] | 673.00 [340.50, 1021.00] | <0.001* |
| Mass, mg | 91.57 [61.10, 172.46] | 349.05 [114.38, 879.92] | <0.001* | 105.78 [60.34, 202.97] | 332.61 [186.64, 767.57] | <0.001* |
| Mean CT Value,Hu | -630.00 [-708.00, -572.00] | -502.00 [-611.00, -346.00] | <0.001* | -619.00 [-678.50, -552.50] | -492.00 [-585.50, -364.00] | <0.001* |
| Standard deviation | 121.14 [94.71, 141.57] | 182.13 [149.47, 211.61] | <0.001* | 136.12 [111.87, 176.21] | 171.99 [145.01, 203.85] | <0.001* |
| Solid Component, % | 0.04 [0.01, 0.21] | 0.27 [0.07, 0.62] | <0.001* | 0.05 [0.02, 0.80] | 1.69 [0.14, 19.40] | <0.001* |
| Sphericity | 0.81 [0.79, 0.83] | 0.79 [0.73, 0.82] | 0.021 | 0.82 [0.79, 0.85] | 0.77 [0.74, 0.81] | <0.001* |
| Energy | 190918206.00 [137210371.00, 324382641.00] | 388371152.00 [129426122.00, 1229293265.00] | 0.010* | 263559738.00 [145259067.00, 389772774.00] | 665376266.00 [225989980.50, 1381011798.00] | 0.001* |
| Entropy | 4.31 [3.97, 4.55] | 4.86 [4.55, 5.20] | <0.001* | 4.52 [4.31, 4.84] | 4.85 [4.56, 5.13] | 0.001* |
| **ΔX_monthly** |  |  |  |  |  |  |
| Diameter | -0.02 [-0.19, 0.00] | 0.00 [-0.29, 0.02] | 0.959 | 0.00 [0.00, 0.02] | 0.00 [0.00, 0.20] | 0.156 |
| Volume | 0.26 [-5.84, 13.89] | -5.02 [-55.10, 2.69] | 0.006* | 4.00 [-1.33, 15.30] | 10.50 [-9.17, 34.00] | 0.605 |
| Surface area | 0.33 [-6.18, 10.83] | -2.02 [-21.76, 5.82] | 0.084 | 4.00 [-2.12, 13.18] | 5.12 [-5.33, 29.50] | 0.779 |
| Mass | 0.42 [-3.58, 8.86] | 0.42 [-17.69, 6.09] | 0.212 | 1.67 [-0.68, 4.45] | 6.67 [-2.14, 18.12] | 0.158 |
| Mean CT value | 4.00 [-5.08, 13.14] | 4.71 [-6.32, 15.19] | 0.931 | -1.25 [-5.05, 2.80] | 1.00 [-4.17, 10.75] | 0.113 |
| Standard deviation | 2.17 [-2.29, 7.08] | 0.49 [-3.32, 5.69] | 0.240 | -1.52 [-5.02, 2.15] | -1.43 [-5.84, 5.89] | 0.396 |
| Solid component | 0.00 [-0.01, 0.02] | 0.01 [-0.01, 0.04] | 0.189 | 0.00 [-0.02, 0.00] | 0.01 [-0.01, 0.38] | 0.035* |
| Sphericity | 0.00 [-0.01, 0.00] | 0.00 [-0.01, 0.00] | 0.259 | 0.00 [-0.01, 0.00] | 0.00 [-0.01, 0.00] | 0.441 |
| Energy | -2092381.42 [-17524885.03, 22014032.15] | -12172017.82 [-52009572.35, 8730157.41] | 0.126 | -5088398.50 [-32746043.49, 11477941.71] | -9101119.92 [-83153051.71, 13834420.74] | 0.540 |
| Entropy | 0.02 [-0.03, 0.11] | 0.01 [-0.04, 0.05] | 0.235 | -0.01 [-0.05, 0.03] | -0.01 [-0.04, 0.04] | 0.286 |
| **X_change_monthly** |  |  |  |  |  |  |
| Diameter | -0.36 [-2.40, 0.00] | 0.00 [-1.84, 0.12] | 0.666 | 0.00 [0.00, 0.20] | 0.00 [0.00, 1.80] | 0.220 |
| Volume | 0.29 [-3.39, 6.32] | -1.43 [-6.27, 0.65] | 0.035* | 1.70 [-0.97, 5.85] | 1.96 [-1.66, 4.82] | 0.859 |
| Surface area | 0.13 [-2.01, 4.04] | -0.63 [-2.20, 1.21] | 0.147 | 1.19 [-0.67, 5.01] | 1.03 [-1.13, 5.99] | 0.954 |
| Mass | 0.69 [-3.27, 8.49] | 0.37 [-3.39, 1.75] | 0.170 | 1.37 [-1.01, 3.78] | 2.08 [-0.83, 4.28] | 0.653 |
| Mean CT value | -0.52 [-2.08, 0.80] | -1.05 [-3.83, 1.17] | 0.501 | 0.22 [-0.43, 0.91] | -0.30 [-2.56, 0.85] | 0.146 |
| Standard deviation | 2.08 [-1.78, 8.92] | 0.20 [-1.67, 3.31] | 0.212 | -0.75 [-4.05, 1.60] | -0.96 [-3.31, 4.06] | 0.344 |
| Solid component | 3.22 [-8.76, 41.02] | 5.16 [-2.78, 20.57] | 0.591 | -1.36 [-6.12, 11.29] | 2.49 [-2.22, 12.25] | 0.105 |
| Sphericity | -0.06 [-0.62, 0.59] | -0.19 [-0.99, 0.29] | 0.265 | -0.11 [-0.85, 0.37] | -0.02 [-0.63, 0.54] | 0.437 |
| Energy | -1.14 [-7.91, 9.20] | -3.67 [-9.49, 4.63] | 0.319 | -2.81 [-12.22, 3.39] | -2.42 [-9.11, 6.64] | 0.653 |
| Entropy | 0.47 [-0.70, 2.27] | 0.19 [-0.77, 1.05] | 0.217 | -0.29 [-1.16, 0.67] | -0.15 [-0.88, 0.76] | 0.275 |
| Data are n (%), n/N (%), or median (IQR). IQR, interquartile range. X_Baseline represents the baseline value of parameter X at the initial CT scan. ΔX denotes the absolute change of parameter X between two time points. ΔX_monthly indicates the absolute rate of change per month, calculated as ΔX divided by follow-up interval time (months). X_change_monthly refers to the relative percentage rate of change per month, calculated as (ΔX / X_B) × 100% divided by follow-up interval time (months).  Abbreviations: ICI, Immune checkpoint inhibitor; RU, right upper lobe; RM, right middle lobe; RL, right lower lobe; LU, left upper lobe; LL, left lower lobe; pGGN, pure ground-glass nodule; PSN, part-solid nodule; VCP, volume change proportion.  *, p < 0.05 was considered statistically significant. | | | | | | |

| Table S4 Characteristics in Lung RADS subgroups between the ICI and control groups. | | | | | | |
| --- | --- | --- | --- | --- | --- | --- |
|  | ICI group | |  | Controls | |  |
|  | Lung RADS < 4A  (n=92) | Lung RADS ≥ 4A  (n=18) | P value | Lung RADS < 4A  (n=76) | Lung RADS ≥ 4A  (n=34) | P value |
| Gender |  |  | 0.613 |  |  | 0.493 |
| Female | 29 (31.5) | 4 (22.2) |  | 22 (28.9) | 7 (20.6) |  |
| Male | 63 (68.5) | 14 (77.8) |  | 54 (71.1) | 27 (79.4) |  |
| Age, years | 63.00 [59.00, 68.00] | 66.00 [60.00, 71.50] | 0.300 | 64.00 [59.00, 69.00] | 62.00 [56.25, 67.75] | 0.424 |
| Smoking History |  |  | 0.879 |  |  | 0.071 |
| No | 61 (66.3) | 11 (61.1) |  | 47 (61.8) | 14 (41.2) |  |
| Yes | 31 (33.7) | 7 (38.9) |  | 29 (38.2) | 20 (58.8) |  |
| Location |  |  | 0.931 |  |  | 0.714 |
| RU | 31(33.7) | 7 (38.9) |  | 39 (51.3) | 13 (38.2) |  |
| RM | 3 (3.3) | 0 (0.0) |  | 5 (6.6) | 3 (8.8) |  |
| RL | 19 (20.7) | 3 (16.7) |  | 7 (9.2) | 4 (11.8) |  |
| LU | 30 (32.6) | 6 (33.3) |  | 17 (22.4) | 8 (23.5) |  |
| LL | 9 (9.8) | 2 (11.1) |  | 8 (10.5) | 6 (17.6) |  |
| Density |  |  | <0.001* |  |  | <0.001* |
| pGGN | 53 (57.6) | 0 (0.0) |  | 46 (60.5) | 1 (2.9) |  |
| PSN | 39 (42.4) | 18 (100.0) |  | 30 (39.5) | 33 (97.1) |  |
| Interval time, months | 5.28 [2.87, 6.80] | 6.02 [3.28, 6.66] | 0.668 | 4.00 [3.00, 8.00] | 3.00 [2.00, 5.50] | 0.032* |
| Lung RADs |  |  | 0.002* |  |  | 0.102 |
| Downgraded | 4 (4.3) | 5 (27.8) |  | 0 (0.0) | 2 (5.9) |  |
| Unchanged | 76 (82.6) | 13 (72.2) |  | 60 (78.9) | 25 (73.5) |  |
| Upgraded | 12 (13.0) | 0 (0.0) |  | 16 (21.1) | 7 (20.6) |  |
| VCP |  |  | 0.462 |  |  | 0.664 |
| Progressive | 23 (25.0) | 3 (16.7) |  | 2 (2.6) | 0 (0.0) | 0.443 |
| Regressive | 52 (56.5) | 13 (72.2) |  | 58 (76.3) | 29 (85.3) |  |
| Stable | 17 (18.5) | 2 (1.1) |  | 16 (21.1) | 5 (14.7) |  |
| **X_Baseline** |  |  |  |  |  |  |
| Diameter, mm | 8.15 [6.64, 12.04] | 14.00 [9.95, 17.64] | <0.001* | 9.00 [7.00, 11.55] | 12.70 [9.05, 16.90] | <0.001* |
| Volume, mm^3^ | 205.00 [127.75, 722.25] | 1577.00 [370.50, 2468.50] | <0.001* | 283.50 [160.75, 671.00] | 1010.00 [335.50, 2311.00] | <0.001* |
| Surface area, mm^2^ | 295.50 [192.75, 610.75] | 965.00 [414.50, 1456.75] | <0.001* | 335.00 [247.50, 645.25] | 749.50 [388.25, 1148.50] | <0.001* |
| Mass, mg | 113.43 [67.56, 352.83] | 632.48 [192.98, 1461.67] | <0.001* | 137.53 [86.10, 311.48] | 581.58 [289.48, 971.00] | <0.001* |
| Mean CT value, Hu | -601.50 [-683.25, -502.75] | -406.00 [-520.00, -302.00] | <0.001* | -585.50 [-659.50, -524.00] | -422.50 [-550.50, -280.75] | <0.001* |
| Standard deviation | 134.77 [107.17, 168.31] | 228.48 [183.75, 257.09] | <0.001* | 146.40 [126.21, 178.30] | 189.59 [150.43, 231.56] | <0.001* |
| Solid Component, % | 0.08 [0.02, 0.30] | 0.45 [0.28, 0.62] | <0.001* | 0.08 [0.03, 2.02] | 6.60 [0.44, 24.38] | <0.001* |
| Sphericity | 0.81 [0.76, 0.83] | 0.75 [0.67, 0.80] | 0.001* | 0.80 [0.77, 0.83] | 0.79 [0.73, 0.82] | 0.030* |
| Energy | 260227054.50 [132615293.50, 520726780.50] | 841237299.00 [282852072.00, 1790954295.25] | 0.011* | 308847755.00 [147145167.50, 782048045.25] | 809687673.50 [297650830.50, 1600785185.25] | 0.003* |
| Entropy | 4.52 [4.11, 4.72] | 5.20 [4.83, 5.48] | <0.001* | 4.67 [4.33, 4.92] | 5.03 [4.62, 5.24] | <0.001* |
| **ΔX_monthly** |  |  |  |  |  |  |
| Diameter | 0.00 [-0.20, 0.00] | -0.06 [-0.64, 0.00] | 0.275 | 0.00 [0.00, 0.09] | 0.00 [0.00, 0.14] | 0.865 |
| Volume | -1.25 [-20.48, 7.29] | -6.77 [-81.70, 2.35] | 0.213 | 4.33 [-2.33, 16.25] | 13.61 [-14.00, 38.31] | 0.457 |
| Surface area | 0.24 [-9.33, 7.89] | -5.02 [-20.95, 4.65] | 0.320 | 5.06 [-4.00, 13.81] | 4.44 [-6.04, 31.66] | 0.872 |
| Mass | 0.42 [-4.95, 8.10] | -0.52 [-48.62, 5.92] | 0.457 | 1.77 [-0.95, 7.03] | 7.23 [-1.43, 22.26] | 0.181 |
| Mean CT value | 4.14 [-5.22, 15.05] | 3.60 [-11.50, 19.87] | 0.790 | -0.69 [-4.45, 7.15] | 0.58 [-2.81, 10.45] | 0.569 |
| Standard deviation | 2.14 [-2.32, 7.73] | -1.17 [-3.61, 0.91] | 0.016 | -0.71 [-5.32, 3.03] | -2.17 [-8.03, 4.14] | 0.866 |
| Solid component | 0.00 [-0.01, 0.02] | 0.01 [-0.01, 0.07] | 0.588 | 0.00 [-0.01, 0.02] | 0.01 [-0.21, 0.05] | 0.587 |
| Sphericity | 0.00 [-0.01, 0.00] | 0.00 [0.00, 0.00] | 0.502 | 0.00 [0.00, 0.00] | 0.00 [-0.01, 0.00] | 0.329 |
| Energy | -2654632.51 [-24548012.35, 20784244.93] | -26451239.83 [-87907550.89, -1573017.21] | 0.043* | -3453346.75 [-34001995.07, 15316908.81] | -26519110.02 [-160351913.00, 10396706.07] | 0.056 |
| Entropy | 0.03 [-0.03, 0.09] | -0.02 [-0.07, 0.02] | 0.027* | -0.01 [-0.05, 0.04] | -0.01 [-0.05, 0.03] | 0.892 |
| **X_change_monthly** |  |  |  |  |  |  |
| Diameter | 0.00 [-2.25, 0.01] | -0.51 [-5.41, 0.00] | 0.504 | 0.00 [0.00, 1.02] | 0.00 [0.00, 1.23] | 0.781 |
| Volume | -0.82 [-4.59, 3.32] | -1.21 [-3.01, -0.05] | 0.611 | 1.63 [-1.36, 5.98] | 2.20 [-1.55, 4.11] | 0.806 |
| Surface area | 0.08 [-2.24, 2.78] | -0.61 [-1.88, 0.96] | 0.443 | 1.20 [-1.24, 5.24] | 0.59 [-0.88, 5.01] | 0.727 |
| Mass | 0.47 [-3.30, 3.55] | -0.26 [-6.22, 2.01] | 0.472 | 1.43 [-0.90, 4.44] | 2.24 [-0.86, 3.55] | 0.841 |
| Mean CT value | -0.73 [-3.03, 0.81] | -0.83 [-3.84, 2.90] | 0.974 | 0.11 [-1.21, 0.86] | -0.10 [-2.34, 0.94] | 0.641 |
| Standard_deviation | 1.50 [-1.64, 6.74] | -0.54 [-2.09, 0.49] | 0.016 | -0.51 [-3.42, 2.84] | -1.29 [-3.63, 2.25] | 0.851 |
| Solid component | 5.40 [-4.66, 34.17] | 2.17 [-4.04, 10.00] | 0.464 | 0.00 [-5.23, 15.72] | 1.52 [-1.98, 6.88] | 0.884 |
| Sphericity | -0.16 [-0.96, 0.50] | 0.20 [-0.55, 0.43] | 0.534 | -0.03 [-0.61, 0.41] | -0.15 [-1.22, 0.37] | 0.335 |
| Energy | -1.20 [-8.38, 7.24] | -3.76 [-9.98, -1.08] | 0.180 | -1.80 [-8.55, 7.17] | -5.19 [-12.73, 3.47] | 0.151 |
| Entropy | 0.55 [-0.59, 1.96] | -0.29 [-1.21, 0.49] | 0.032* | -0.22 [-1.15, 0.84] | -0.21 [-0.93, 0.64] | 0.907 |
| Data are n (%), n/N (%), or median (IQR). IQR, interquartile range. X_Baseline represents the baseline value of parameter X at the initial CT scan. ΔX denotes the absolute change of parameter X between two time points. ΔX_monthly indicates the absolute rate of change per month, calculated as ΔX divided by follow-up interval time (months). X_change_monthly refers to the relative percentage rate of change per month, calculated as (ΔX / X_B) × 100% divided by follow-up interval time (months).  Abbreviations: ICI, Immune checkpoint inhibitor; RU, right upper lobe; RM, right middle lobe; RL, right lower lobe; LU, left upper lobe; LL, left lower lobe; pGGN, pure ground-glass nodule; PSN, part-solid nodule; VCP, volume change proportion.  *, p < 0.05 was considered statistically significant. | | | | | | |

| Table S5 Comparison of histologically confirmed GGNs between the ICI group and control group. | | | |
| --- | --- | --- | --- |
| Variables | ICI group (n=5) | Controls (n=11) | p value |
| Gender |  |  | 0.036* |
| Female | 4 (80.0%) | 2 (18.2%) |  |
| Male | 1 (20.0%) | 9 (81.8%) |  |
| Age, years | 61.00 [61.00, 63.00] | 64.00 [56.00, 70.00] | 0.4 |
| Smoking history |  |  | 0.12 |
| No | 5 (100.0%) | 6 (54.5%) |  |
| Yes | 0 (0.0%) | 5 (45.5%) |  |
| Location |  |  | 0.7 |
| RU | 2 (40.0%) | 3 (27.3%) |  |
| RM | 0 (0.0%) | 2 (18.2%) |  |
| RL | 2 (40.0%) | 1 (9.1%) |  |
| LU | 1 (20.0%) | 4 (36.4%) |  |
| LL | 0 (0.0%) | 1 (9.1%) |  |
| Histology |  |  | >0.9 |
| IAC | 4 (80.0%) | 7 (63.6%) |  |
| MIA | 1 (20.0%) | 4 (36.4%) |  |
| Density |  |  | >0.9 |
| pGGN | 1 (20.0%) | 4 (36.4%) |  |
| PSN | 4 (80.0%) | 7 (63.6%) |  |
| Interval time, months | 4.80 [3.40, 4.80] | 4.00 [3.00, 8.00] | 0.8 |
| Baseline Lung RADS |  |  | 0.2 |
| 2 | 1 (20.0%) | 5 (45.5%) |  |
| 3 | 3 (60.0%) | 2 (18.2%) |  |
| 4A | 0 (0.0%) | 3 (27.3%) |  |
| 4B | 1 (20.0%) | 0 (0.0%) |  |
| 4X | 0 (0.0%) | 1 (9.1%) |  |
| Lung RADs change |  |  | >0.9 |
| Unchanged | 3 (60.0%) | 6 (54.5%) |  |
| Upgraded | 2 (40.0%) | 5 (45.5%) |  |
| VCP |  |  | 0.15 |
| Regressive | 2 (40.0%) | 0 (0.0%) |  |
| Stable | 2 (40.0%) | 8 (72.7%) |  |
| Progressive | 1 (20.0%) | 3 (27.3%) |  |
| **X_Baseline** |  |  |  |
| Diameter, mm | 9.30 [7.65, 11.50] | 9.00 [7.00, 13.00] | 0.7 |
| Volume, mm^3^ | 206.00 [203.00, 709.00] | 356.00 [174.00, 648.00] | >0.9 |
| Surface area, mm^2^ | 272.00 [228.00, 524.00] | 413.00 [204.00, 738.00] | >0.9 |
| Mass, mg | 170.79 [164.04, 468.15] | 294.74 [101.74, 437.32] | 0.8 |
| Mean CT value, Hu | -400.00 [-539.00, -346.00] | -503.00 [-629.00, -283.00] | >0.9 |
| Standard deviation | 153.17 [149.73, 177.95] | 170.69 [133.08, 190.61] | >0.9 |
| Solid component, % | 0.21 [0.11, 0.42] | 0.38 [0.04, 18.27] | 0.6 |
| Sphericity | 0.82 [0.74, 0.86] | 0.81 [0.73, 0.84] | 0.7 |
| Energy | 89,694,970.00[85,703,912.00, 292,241,517.00] | 378,062,091.00[117,925,333.00, 1,326,195,707.00] | 0.3 |
| Entropy | 4.67 [4.60, 4.81] | 4.78 [4.38, 5.26] | >0.9 |
| **ΔX_monthly** |  |  |  |
| Diameter | -0.04 [-0.13, 0.11] | 0.00 [0.00, 0.25] | 0.3 |
| Volume | 3.33 [-12.92, 12.50] | 21.33 [6.75, 33.67] | 0.054 |
| Surface area | 7.32 [-5.21, 10.83] | 7.33 [1.63, 25.00] | 0.7 |
| Mass | 17.05 [8.86, 30.29] | 8.95 [4.79, 35.10] | 0.8 |
| Mean CT value | 13.33 [4.17, 18.45] | -1.08 [-22.00, 5.33] | 0.14 |
| Standard deviation | 1.77 [1.05, 13.83] | -2.35 [-13.07, 0.07] | 0.041* |
| Solid component | 0.02 [0.02, 0.02] | 0.00 [-0.07, 1.52] | 0.2 |
| Sphericity | -0.01 [-0.01, 0.00] | 0.00 [-0.01, 0.00] | 0.8 |
| Energy | 3,098,928.13[-4,507,281.27, 50,184,826.04] | -11,292,793.67[-66,170,574.67, 2,881,665.00] | 0.3 |
| Entropy | 0.08 [0.05, 0.12] | -0.02 [-0.04, 0.03] | 0.07 |
| **X_change_monthly** |  |  |  |
| Diameter | -0.57 [-1.36, 1.00] | 0.00 [0.00, 2.64] | 0.2 |
| Volume | 0.07 [-6.27, 1.76] | 5.41 [1.74, 13.22] | 0.07 |
| Surface area | 2.07 [-0.59, 2.69] | 2.19 [0.40, 6.86] | 0.7 |
| Mass | 6.37 [1.89, 10.39] | 2.92 [1.37, 12.47] | >0.9 |
| Mean CT value | -4.40 [-4.74, -1.04] | 0.17 [-1.33, 9.04] | 0.14 |
| Standard deviation | 1.15 [0.59, 11.42] | -1.38 [-7.03, 0.04] | 0.041* |
| Solid component | 8.68 [8.25, 18.93] | -3.52 [-14.66, 5.22] | 0.041* |
| Sphericity | -0.62 [-1.57, 0.43] | -0.25 [-1.38, 0.25] | 0.8 |
| Energy | 4.64 [-3.69, 17.17] | -3.79 [-12.08, 3.78] | 0.11 |
| Entropy | 1.64 [1.00, 2.50] | -0.55 [-0.76, 0.57] | 0.089 |
| Data are n (%), n/N (%), or median (IQR). IQR, interquartile range. X_Baseline represents the baseline value of parameter X at the initial CT scan. ΔX denotes the absolute change of parameter X between two time points. ΔX_monthly indicates the absolute rate of change per month, calculated as ΔX divided by follow-up interval time (months). X_change_monthly refers to the relative percentage rate of change per month, calculated as (ΔX / X_B) × 100% divided by follow-up interval time (months).  Abbreviations: ICI, Immune checkpoint inhibitor; RU, right upper lobe; RM, right middle lobe; RL, right lower lobe; LU, left upper lobe; LL, left lower lobe; MIA, minimally invasive adenocarcinoma ; IAC, invasive adenocarcinomas; pGGN, pure ground-glass nodule; PSN, part-solid nodule; VCP, volume change proportion.  *, p < 0.05 was considered statistically significant. | | | |


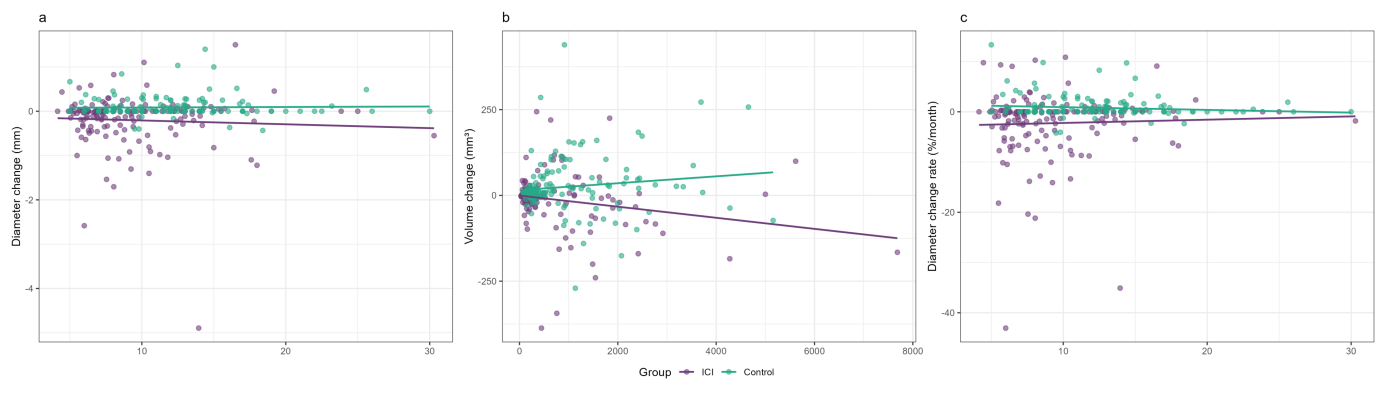


Figure S1 Relationship between the absolute monthly change rates in diameter(a) and volume(b), and the relative monthly change rates in diameter(c), and their corresponding baseline values from the mixed-effects model analysis.


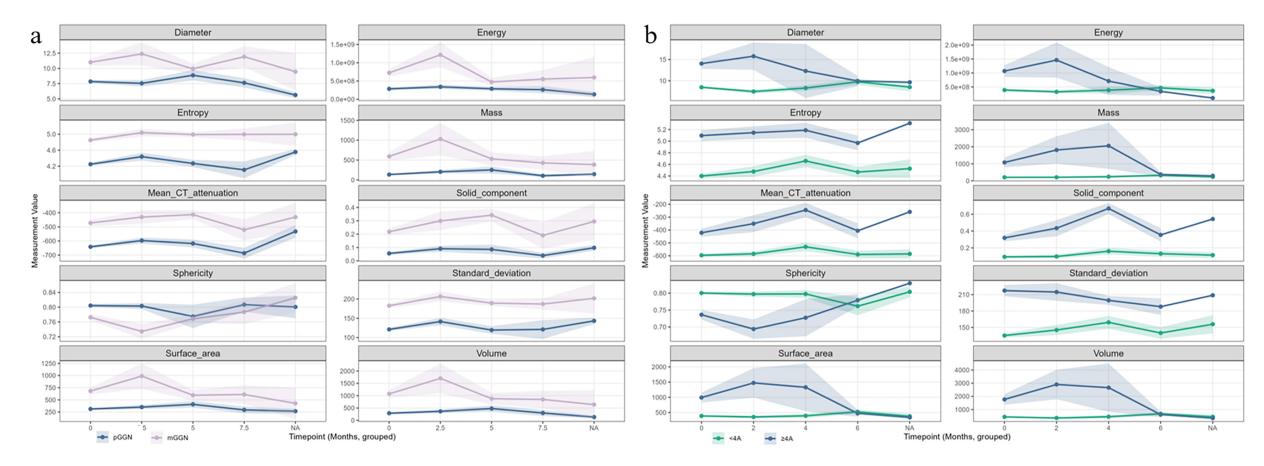


Figure S2 Subgroup trends of imaging parameter changes during follow-up within the density (a) and Lung-RADS (b) subgroups in the ICI group.
